# Healthcare utilization for common infectious disease syndromes in Soweto and Klerksdorp, South Africa

**DOI:** 10.11604/pamj.2018.30.271.14477

**Published:** 2018-08-10

**Authors:** Karen Kai-Lun Wong, Claire von Mollendorf, Neil Martinson, Shane Norris, Stefano Tempia, Sibongile Walaza, Ebrahim Variava, Meredith Lynn McMorrow, Shabir Madhi, Cheryl Cohen, Adam Lauren Cohen

**Affiliations:** 1 Centers for Disease Control and Prevention, Atlanta, Georgia USA; 2 United States Public Health Service; 3 National Institute for Communicable Diseases, Johannesburg, South Africa; 4 University of Witwatersrand, Johannesburg, South Africa; 5 MRC Developmental Pathways for Health Research Unit, University of Witwatersrand, Johannesburg, South Africa; 6 Johns Hopkins University, Baltimore, Maryland USA; 7 Klerksdorp-Tshepong Hospital Complex, Klerksdorp, South Africa

**Keywords:** Diarrhea, health services, meningitis, respiratory tract infections, South Africa

## Abstract

**Introduction:**

Understanding healthcare utilization helps characterize access to healthcare, identify barriers and improve surveillance data interpretation. We describe healthcare-seeking behaviors for common infectious syndromes and identify reasons for seeking care.

**Methods:**

We conducted a cross-sectional survey among residents in Soweto and Klerksdorp, South Africa. Households were interviewed about demographic characteristics; recent self-reported episodes of pneumonia, influenza-like illness (ILI), chronic febrile respiratory illness and meningitis in individuals of all ages; recent diarrhea in children aged < 5 years; and consultation with healthcare facilities and providers.

**Results:**

From July-October 2012, we interviewed 1,442 households in Klerksdorp and 973 households in Soweto. Public clinics were consulted most frequently for pneumonia, ILI and diarrhea in a child <5 years old at both sites; public hospitals were most frequently consulted for chronic respiratory and meningitis syndromes. Of all illness episodes reported, there were 110 (35%) in Klerksdorp and 127 (32%) in Soweto for which the person did not seek care with a licensed medical provider. Pharmacies were often consulted by individuals with pneumonia (Klerksdorp: 17, 16%; Soweto: 38, 22%) or ILI (Klerksdorp: 35, 24%; 44, 28%). Patients who did not seek care with a licensed provider reported insufficient time (Klerksdorp: 7%; Soweto, 20%) and lack of medications at the facility (Klerksdorp: 4%; Soweto: 8%) as barriers.

**Conclusion:**

Public government healthcare facilities are commonly consulted for infectious syndromes and pharmacies are frequently consulted particularly for respiratory diseases. Improving medication availability at healthcare facilities and streamlining healthcare delivery may improve access of licensed providers for serious illnesses.

## Introduction

Sentinel surveillance for severe acute respiratory illness (SARI), influenza-like illness (ILI), diarrhea and meningitis at selected healthcare facilities in South Africa is important for defining the epidemiology and scope of common infectious morbidities, identifying etiologies [1, 2], describing seasonality of infectious syndromes [3] and characterizing emerging pathogens [4-6]. However, data from sentinel surveillance should be interpreted within the context of healthcare utilization patterns of the community, as people may consult with a range of licensed medical providers as well as pharmacies, traditional healers and religious leaders for certain illnesses. People may not seek healthcare through clinics and hospitals because of economic or logistical barriers [7, 8], negative perceptions of the healthcare system [9], and limited insight into illness and the need for care [7]. Barriers to healthcare may differ regionally [10, 11], underscoring the need for region-specific healthcare utilization and accessibility data. Understanding reasons for not seeking care helps the medical community and health policymakers improve healthcare delivery. Medical care in South Africa is available through public government facilities; the private sector health system, complementary and traditional medicine practitioners, pharmacies and self-care are additional healthcare options [12, 13]. Previous studies found that some patients with chronic illnesses in South Africa do not seek healthcare at all [8, 9]. Healthcare utilization surveys have been conducted in several countries to complement sentinel surveillance systems by providing a context of healthcare-seeking behaviors for the catchment population of a sentinel site [14-16]. We describe healthcare-seeking behaviors for common infectious syndromes in an urban and a peri-urban site to inform current surveillance efforts, characterize the population seeking healthcare in the community and identify reasons underlying decisions to seek care.

## Methods

**Study area and population:** The survey was conducted at two sites in South Africa from July to October 2012, near the end of the typical influenza season [3]. Soweto is an urban township outside Johannesburg, Gauteng Province, with a population of approximately 1.3 million in 2011 [17]. The average household income for Soweto is estimated at ZAR 6500 (USD 606) [18, 19]. Soweto is served by a large public hospital (Chris Hani Baragwanath Academic Hospital [CHBAH]), a sentinel site for SARI surveillance [20]. Residents of Soweto also access other clinics, private general practitioners and other providers. CHBAH is a secondary-tertiary care hospital. Adults who present to CHBAH with uncomplicated illness requiring level one or two care are automatically referred to Selby Hospital, a lower care-level hospital. Those with non-urgent issues are referred to a primary care clinic. The prevalence of HIV in the City of Johannesburg was 11.1% in 2012 [21] and among pregnant women in the district it was 28.9% in 2011 [22]. The Klerksdorp study site encompasses peri-urban townships surrounding Klerksdorp in the local municipality of Matlosana in the Dr Kenneth Kaunda District of North West Province. Townships in the Klerksdorp study site included Jouberton, Alabama, Sakhrol, Kanana, Khuma, Tigane, Dominionville and Vaal Reefs, for a combined population of more than 274,000 in 2011 [17]. Residents of the Klerksdorp site townships are served by the public Klerksdorp/Tshepong Hospital Complex (KTHC), comprising Klerksdorp Hospital and Tshepong Hospital. KTHC is a SARI sentinel surveillance site [20] and a referral hospital. Hospital emergency services are reserved for emergent issues; non-urgent issues are referred to a primary care clinic. HIV prevalence among pregnant women in the district was estimated at 36% in 2011 [22] and prevalence in the general population in North West province was 13.3% in 2012 [21]. In South Africa, healthcare is available at public government outpatient clinics and hospitals for little or no cost. Private outpatient clinics, private hospitals, and general practitioners also offer services for a fee. In this study, the term "general practitioner" refers to individuals in private practice and may include general practitioners and specialists. In 2015, nearly a quarter of South African households included at least one member belonging to a medical aid scheme. Traditional healers and religious leaders, including spiritual healers, faith healers, or prophets, provide alternative or complementary medicine services for a fee.

**Study design and geographic sampling methods:** We conducted a cross-sectional survey of households at each of the two study sites using a one-stage cluster design similar to methods used by Lindblade et al [14]. The boundaries of residential areas at each site were determined using Google Earth 6.0 and verified by residents of each site. Areas that were likely non-residential, such as parks and schools, were excluded if non-residential status could be verified by Google Street View or by a local resident. A simple random sample of geographic coordinates (GeoIDs (Geographic Identification number)) within the residential areas was generated using the cruise points function of the KML Tools Project with a buffer of 30 meters around each point [23]. A multilingual team of trained interviewers navigated to each GeoID using a handheld global positioning system (GPS) device and selected the nearest household within 30 meters to interview. If no dwelling existed within 30 meters of the GeoID, the GeoID was considered invalid. Teams visited households up to three times on separate days and times to enumerate all members of the household including those who had died in the last year. This team interviewed household members or their surrogates about demographic characteristics, medical conditions that may influence healthcare seeking, selected illnesses and healthcare utilization using a standardized questionnaire. Participants were asked to report all locations and providers consulted for each illness and the order in which they were consulted. Interviews were conducted in the preferred language of the household, including English, Xhosa, Setswana, or Zulu.

**Sample size:** The sample size was calculated to estimate healthcare utilization for pneumonia with 95% confidence intervals and 10% precision. The cumulative incidence of pneumonia was assumed to be 2% per year based on other healthcare utilization surveys [14, 24] and at least 50% of pneumonia patients were assumed to seek healthcare. The non-response rate was assumed to be 15% and the household size was assumed to be 3.5. Based on these assumptions, we aimed to visit at least 1614 households at each of the sites. We assumed some geographic coordinates would not correspond to a household and that this would happen more in Soweto where residential boundaries are less clear; thus, approximately 100 extra coordinates in Soweto and 50 extra coordinates in Klerksdorp were generated to account for those that may not correspond to a household. We sampled 1713 coordinates in Soweto, and 1669 coordinates in Klerksdorp.

**Selected illnesses surveyed and case definitions:** The interview team described selected infectious syndromes and classified illness reported by participants according to the following case definitions: a) Pneumonia, defined as sudden onset or worsening fever (temperature > 38°C or subjective) and cough and difficult breathing lasting 2-30 days or as diagnosed by a healthcare worker within the last year in a person of any age; ILI, defined as sudden onset fever or worsening fever (T > 38°C or subjective) with cough and/or sore throat within the last 30 days in a person of any age. b) Chronic febrile respiratory illness, defined as fever and cough and either difficult breathing or weight loss lasting ≥ 30 days within the last year in a person of any age. c) Diarrhea in a child, defined by ≥3 loose or watery stools within a 24 hour period within the last 14 days in a child < 5 years of age, or d) Meningitis, defined by fever or headache and one of the following: stiff neck, confusion, new weakness in arm or leg, or double vision, within the last year, in a person of any age. Case definitions for pneumonia, ILI and diarrhea were based on those used in other healthcare utilization surveys [14, 16, 24-27]. The criteria for chronic febrile respiratory illness were selected to be consistent with symptom screening tests for tuberculosis [28]. The meningitis case definition, while non-specific for bacterial meningitis, was designed to be sensitive for syndromes consistent with either acute or chronic meningitis. For each illness, information was collected on all types of healthcare services sought and the services obtained. Licensed medical providers were defined as any of the following: public clinics, public hospitals, private clinics, private hospitals, or general practitioners. Additional analysis was performed for severe pneumonia. Episodes of non-fatal pneumonia that reported in the same month of the interview were excluded from this analysis, based on findings by Burton et al [26] in Kenya that episodes of respiratory illness reported within two weeks of the interview were more likely to be milder respiratory illnesses. Severe pneumonia was defined as follows: a) Non-fatal cases: among patients < 3 years old, any illness meeting pneumonia criteria plus one of the following: admitted to hospital, unable to drink or breastfeed, vomits everything, unconscious, convulsions, difficulty with breathing or fast breathing, blue mouth or fingers; among patients ≥ 3 years old, any illness meeting pneumonia criteria plus one of the following: admitted to hospital, confusion, convulsions, unconscious, vomits everything, unable to get up from bed. b) Fatal cases: any illness meeting criteria for pneumonia.

**Ethical considerations:** The purpose of the study was explained to an adult primary caregiver, identified by the household as the person most involved in the daily care of the household members, in the preferred language of the caregiver. Participating caregivers provided written consent on behalf of all household members to provide household-level data. For each household member aged ≥18 years on whom individual data were collected, verbal consent was obtained from the individual. If that person was unavailable, the primary caregiver verbally consented on behalf of the unavailable individual. For persons aged < 18 years, the primary caregiver provided information on behalf of that individual. The study was determined to be within the scope of public health practice by the U.S. Centers for Disease Control and Prevention. The study protocol was also reviewed and approved by the University of the Witwatersrand human research ethics committee (M120367).

**Statistical analysis:** Taylor linearization was used for variance estimation to account for clustering of illnesses by household and by individual. Analysis was conducted using SAS 9.3 (Cary, NC) PROC SURVEYFREQ for bivariate analyses and PROC SURVEYLOGISTIC for multivariable analyses. Variables with a significance of p < 0.2 were selected for inclusion in the multivariable model of factors associated with seeking care with licensed medical providers. We used the Rao-Scott chi-square test to evaluate differences between two or more proportions. All p-values are two-sided and p < 0.05 was considered significant. Maps were created using R 3.0.1 [29], with packages sp [30, 31] and rgdal [32].

## Results

**Figure 1:**
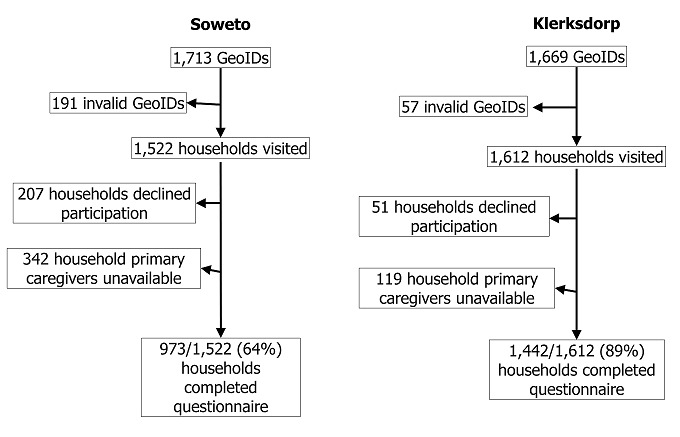
healthcare utilization survey participant enrollment, in Soweto and Klerksdorp, South Africa, August–September 2012

There were 1,713 coordinates visited in Soweto, of which 191 (11%) were not within 30 meters of a dwelling and were considered invalid. In Klerksdorp, 1,669 geographic coordinates were visited, of which 57 (3%) were invalid. Of the 1,522 households visited in Soweto, there were 549 (36%) households that did not complete the questionnaire, including 207 (38%) that declined participation and 342 (62%) for which the primary caregiver was unavailable. In Klerksdorp, 170 (11%) of the 1,612 households visited did not complete the questionnaire, including 51 (30%) that declined participation and 119 (70%) for which the primary caregiver was unavailable. There were 973 households in Soweto and 1,442 households in Klerksdorp that completed the questionnaire (Figure 1). Most households in Soweto and Klerksdorp reported a household monthly income between ZAR 1,000 and ZAR 2,000 (118-237 USD [18]) (Table 1), and the median household size was four. There were 6,097 individuals surveyed in Klerksdorp and 4,364 individuals in Soweto. Almost half of household members were male (Klerksdorp: 2795, 46%; Soweto: 1999, 46%). Of those whose underlying medical conditions were known, the most prevalent reported medical condition was hypertension (Klerksdorp: 254, 9%; Soweto: 175, 9%). Prevalence of self-reported HIV infection was 6% (334/5731) in Klerksdorp and 3% (113/4142) in Soweto. There were 56 (1%) individuals from Klerksdorp and 29 (1%) individuals from Soweto included who had died within the last year. There were 116 (2%) episodes of pneumonia reported among 6,097 individuals in Klerksdorp and 176 (4%) reported among 4,364 individuals in Soweto. Prevalence of ILI within the last 30 days was 2% (143/6,097) in Klerksdorp and 4% (156/4,364) in Soweto. Symptoms of chronic respiratory disease in the last year were reported for 24 (< 1%) individuals in Klerksdorp and 26 (1%) individuals in Soweto. Diarrhea in the last 2 weeks among children < 5 years of age was reported for 5% (33/725) of children in Klerksdorp and 4% (20/513) of children in Soweto. Meningitis symptoms in the last year were reported for 38 (1%) of individuals in Klerksdorp and 27 (1%) of individuals in Soweto. The proportion of individuals experiencing any infectious syndrome (pneumonia, ILI, chronic respiratory illness, diarrhea in a child < 5 years of age, meningitis) was higher among individuals in Soweto compared with individuals in Klerksdorp (9% versus 5%; p < 0.01). There were 25 (0.4%) in Klerksdorp and 14 (0.3%) in Soweto who reported experiencing > 1 infectious syndrome.

**Table 1:** Characteristics of households and enrolled members by site, in Soweto and Klerksdorp, South Africa, 2012

Characteristic	Klerksdorp	Soweto
No. of households	N=1,442	N=973
**Monthly household income, n (%) ^a^**		
<ZAR 500 (<59 USD)	128 (9)	91 (9)
ZAR 500 to <1,000 (59–118 USD)	236 (16)	162 (17)
ZAR 1,000 to <2,000 (118–237 USD)	536 (37)	285 (29)
ZAR 2,000 to <5,000 (237–592 USD)	241 (17)	150 (15)
ZAR 5,000 to <10,000 (592–1183 USD)	134 (9)	67 (7)
ZAR 10,000 to <15,000 (1183–1775 USD)	21 (1)	22 (2)
≥ZAR 15,000 (>1775 USD)	8 (1)	25 (3)
Don’t know	46 (3)	100 (10)
No response	92 (6)	71 (7)
Median household size (IQR)	4 (3–5)	4 (3–5)
No. of individuals surveyed	N=6,097	N=4,364
Male^b^, n (%)	2,795 (46)	1,999 (46)
**Age group^c^, n (%)**		
<2y	242 (4)	177 (4)
2-4y	466 (8)	270 (6)
5-17y	1,658 (27)	955 (22)
18-64y	3,476 (57)	1,656 (39)
65y+	238 (4)	240 (6)
**Underlying medical conditions, n/N (%)**		
Asthma	107/6,037 (2)	112/4,314 (3)
Diabetes	112/6,009 (2)	145/4,291 (3)
HIV	334/5,731 (6)	113/4,142 (3)
Heart disease	89/6,019 (1)	80/4,276 (2)
Pregnancy, among women 13–49 years	64/1,895 (3)	45/1,334 (3)
Tuberculosis	94/6,004 (2)	36/4,281 (1)
Hypertension	254/2,779 (9)	175/1,999 (9)
**Infectious syndromes, n (%)**		
None reported	5,782 (95)	3,981 (91)
≥1 infectious syndrome reported	315 (5)	383 (9)
Pneumonia	116 (2)	176 (4)
ILI	143 (2)	156 (4)
Chronic respiratory disease	24 (<1)	26 (1)
Diarrhea^d^	33 (5)	20 (4)
Meningitis	38 (1)	27 (1)
Died within last 1 year	56 (1)	29 (1)

a ZAR to USD conversions as of September 1, 2012 [18]

b Percent reported out of 6,075 individuals in Klerksdorp and 4,360 individuals in Soweto with non-missing records

c Percent reported out of 6,080 individuals in Klerksdorp and 4,298 individuals in Soweto with non-missing records

d Includes 9 in Klerksdorp and 22 in Soweto who report being diagnosed with pneumonia by a healthcare worker

e Percent reported out of 725 individuals in Klerksdorp and 513 individuals in Soweto <5 years of age

Overall, use of healthcare facilities was similar between individuals in Soweto and Klerksdorp (Table 2). Public clinics were the facility consulted most frequently for pneumonia, ILI, and diarrhea in a child < 5 years old at both sites; public hospitals were the most frequently consulted for chronic respiratory and meningitis syndromes. Consultation with traditional healers and religious leaders was less frequent in both Soweto and Klerksdorp. Pharmacies were often consulted by individuals with pneumonia (Klerksdorp: 17, 16%; Soweto: 38, 22%) or ILI (Klerksdorp: 35, 24%; 44, 28%). Of all illness episodes, there were 110 (35%) in Klerksdorp and 127 (32%) in Soweto for which the person did not seek care with a licensed medical provider. In Klerksdorp, there were no significant differences by age or by gender in the place where healthcare was sought. In Soweto, children < 5 years old were more likely to seek care at a private clinic or hospital than children ≥ 5 years old (19% versus 7%, RR 2.6, 95% CI 1.4-4.8) and they were less likely to seek care at a pharmacy (12% versus 27%, RR 0.4, 95% CI 0.2-0.9). At both sites, persons with ILI were less likely to seek care at a public clinic or hospital than those with other illnesses (Klerksdorp: RR 0.8, 95% CI 0.6-1.0; Soweto: RR 0.6, 95% CI 0.4-0.8), and those with meningitis symptoms were more likely to seek care at a public clinic or hospital than those with other illnesses (Klerksdorp: RR 2.0, 95% CI 1.6-2.4; Soweto: RR 1.7, 95% CI 1.3-2.3). Public clinics were the most common first place to seek care; there was no difference in this observation by age group (< 5 or ≥ 5 years old). Public clinics were the most common first place to seek care for all infectious syndromes. However, some patients with ILI sought care first at a pharmacy (Klerksdorp: 28%, Soweto: 26%). In Klerksdorp, HIV-infected individuals were more likely to seek care with a licensed medical provider for illness than HIV-negative individuals (adjusted odds ratio [aOR]: 2.8; 95% CI: 1.1-7.2; p = 0.03 in multivariable analysis) (Table 3). In Soweto, HIV-infected individuals also more often sought care with a licensed medical provider (OR: 1.8; 95% CI: 0.7-4.9, p = 0.2), although this difference was not statistically significant. In Soweto, characteristics significantly associated with seeking care in the multivariable analysis included female sex (aOR: 2.0; 95% CI: 1.2-3.3; p = 0.01) and age < 18 years (aOR: 2.5; 95% CI: 1.4-4.4; p = 0.002). Among illnesses for which the individual did not seek care with a licensed medical provider (Klerksdorp: 110; Soweto: 127), the most common reason cited was that the patient was not sick enough (Klerksdorp: 37, 34%; Soweto: 49, 39%) (Supplementary Table 4). Perceived deficiencies in medical supplies or service were also common reasons for not seeking care, including beliefs that the clinic did not have medication, that treatment was not effective, and that there were long queues at the clinic. Patients also cited limited personal resources and logistical issues as reasons for not seeking care, including not having time (Klerksdorp: 8, 7%; Soweto: 26, 20%), transportation difficulties (Klerksdorp: 2, 2%; Soweto: 4, 3%), expense (Klerksdorp: 4, 4%; Soweto: 3, 2%) and inability to arrange childcare (Klerksdorp: 1, 1%; Soweto: 3, 2%). There were no individuals who reported that they did not know where to go or whom to ask about seeking care.

**Table 2:** All healthcare facilities and providers consulted by persons reporting infectious syndromes^a^, in Soweto and Klerksdorp, South Africa, 2011–2012

	All syndromes		Pneumonia		ILI		Chronic respiratory		Diarrhea		Meningitis	
	Kl	So	Kl	So	Kl	So	Kl	So	Kl	So	Kl	So
	n=317	n=394	n=108	n=174	n=143	n=156	n=17	n=24	n=32	n=20	n=27	n=25
Public clinic	122 (38)	157 (40)	42 (39)	75 (43)	56 (39)	52 (33)	5 (29)	10 (42)	13 (41)	11 (55)	6 (22)	9 (36)
Public hospital	69 (22)	63 (16)	30 (28)	29 (17)	9 (6)	9 (6)	15 (88)	13 (54)	3 (9)	1 (5)	22 (81)	14 (56)
Private clinic	29 (9)	45 (11)	13 (12)	17 (10)	10 (7)	23 (15)	2 (12)	2 (8)	4 (13)	3 (15)	0 (0)	0 (0)
Private hospital	7 (2)	21 (5)	1 (1)	11 (6)	4 (3)	9 (6)	0 (0)	1 (4)	0 (0)	0 (0)	2 (7)	2 (8)
General practitioner	39 (12)	53 (13)	15 (14)	21 (12)	16 (11)	28 (18)	3 (18)	2 (8)	4 (13)	2 (10)	1 (4)	0 (0)
Pharmacy	56 (18)	89 (23)	17 (16)	38 (22)	35 (24)	44 (28)	1 (6)	3 (13)	2 (6)	2 (10)	1 (4)	2 (8)
Traditional healer	5 (2)	1 (0)	1 (1)	1 (1)	2 (1)	0 (0)	1 (6)	0 (0)	1 (3)	0 (0)	0 (0)	0 (0)
Religious leader	4 (1)	5 (1)	1 (1)	2 (1)	2 (1)	2 (1)	1 (6)	0 (0)	0 (0)	0 (0)	0 (0)	1 (4)
Health volunteer	2 (1)	6 (2)	1 (1)	4 (2)	1 (1)	2 (1)	0 (0)	0 (0)	0 (0)	0 (0)	0 (0)	0 (0)
Friend	4 (1)	10 (3)	0 (0)	2 (1)	2 (1)	6 (4)	0 (0)	0 (0)	2 (6)	0 (0)	0 (0)	2 (8)
Other	21 (7)	30 (8)	3 (3)	8 (5)	15 (10)	17 (11)	1 (6)	1 (4)	2 (6)	4 (20)	0 (0)	0 (0)
Did not seek care with licensed provider ^b^	110 (35)	127 (32)	30 (28)	56 (32)	0 (0)	0 (0)	64 (45)	62 (40)	1 (6)	1 (4)	15 (47)	5 (25)

Abbreviations: ILI, influenza-like illness; Kl, Klerksdorp; So, Soweto

a ≥1 facility or provider could be consulted

b Licensed medical providers defined as public or private clinics, hospitals, and general practitioners

**Table 3:** household and individual characteristics among living individuals associated with seeking care with a licensed medical provider^a^, in Soweto and Klerksdorp, South Africa, 2011–2012

	Klerksdorp			Soweto		
Characteristic	Sought care, n/N (%)	OR (95% CI)	aOR (95% CI)	Sought care, n/N (%)	OR (95% CI)	aOR (95% CI)
**Monthly household income ^b^**						
< ZAR 2000						
(<237 USD)	107/175 (61)	0.7 (0.4–1.2)	0.6 (0.3–1.2)	150/211 (71)	1.6 (1–2.6)	1.4 (0.8–2.4)
≥ ZAR 2000						
(≥237 USD)	56/80 (70)			66/109 (61)		
**Household size**						
≥ 4	119/190 (63)	1.1 (0.7–1.9)	*	189/277 (68)	1.5 (0.9–2.4)	0.9 (0.6–1.5)
< 4	57/96 (59)			56/95 (59)		
Female	114/178 (64)	1.3 (0.8–2.2)	*	152/220 (69)	1.4 (0.9–2.2)	2.0 (1.2–3.3)
Male	62/108 (57)			92/151 (61)		
<18 y	70/116 (60)	0.9 (0.6–1.5)	*	98/130 (75)	2 (1.2–3.2)	2.5 (1.4–4.4)
≥ 18 y	106/170 (62)			145/240 (60)		
HIV-infected	28/34 (82)	3.2 (1.2–8.1)	2.8 (1.1–7.2)	17/22 (77)	1.8 (0.7–4.9)	*
HIV-uninfected	140/235 (60)			217/334 (65)		
**Household owns cell phone**						
Yes	161/263 (61)	0.8 (0.3–2.1)	*	232/357 (65)	0.3 (0.1–1.3)	0.1 (0.01–1.2)
No	15/23 (65)			13/15 (87)		
**Household owns vehicle**						
Yes	20/40 (50)			67/111 (60)	0.7 (0.4–1.1)	0.8 (0.5–1.5)
		No	156/246 (63)	178/261 (68)		

a Licensed medical providers defined as public or private clinics, hospitals, and general practitioners.

b ZAR to USD conversions as of September 1, 2012 [18]

* Variables with p>0.2 in bivariate analysis excluded from multivariable mode

**Table 4:** Reasons cited by living patient or patient’s caretaker for not seeking care with a licensed medical provider^a^ for an illness, in Soweto and Klerksdorp, South Africa, 2011–2012

Reason	Klerksdorp	Soweto
	n=110	n=127
Not sick enough	37 (34)	49 (39)
Not enough time/unable to take time from work	8 (7)	26 (20)
No medications at clinic/hospital	4 (4)	10 (8)
Too expensive	4 (4)	3 (2)
Dislikes going to clinic/hospital	3 (3)	7 (6)
Treatment is not effective	3 (3)	8 (6)
Long queues	5 (5)	7 (6)
Transportation difficulties	2 (2)	4 (3)
No one available to look after children/house	1 (1)	3 (2)
Did not know where to go/whom to ask	0	0
Other	6 (5)	3 (2)
Don’t know/no response	16 (15)	5 (4)

a Licensed medical providers defined as public or private clinics, hospitals, and general practitioners

**Figure 2:**
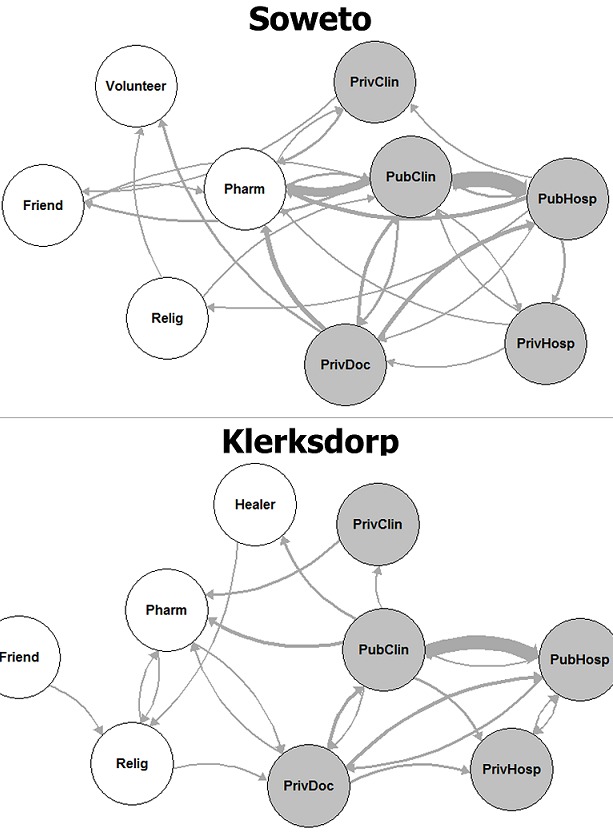
Frequency and direction of flow for individuals seeking care at multiple facilities for infectious syndromes, South Africa 2011–2012 (Soweto: n = 52; Klerksdorp: n = 41); PubHosp: public hospital; PrivHosp: private hospital; Pubclin: public clinic; PrivClin: private clinic; PrivDoc: private doctor or general practitioner; Pharm: pharmacy; Relig: religious leader; Healer, traditional healer; edge thicknesses are proportional to frequency to movement between facilities; shaded circles indicate facilities defined as licensed medical providers

**Figure 3:**
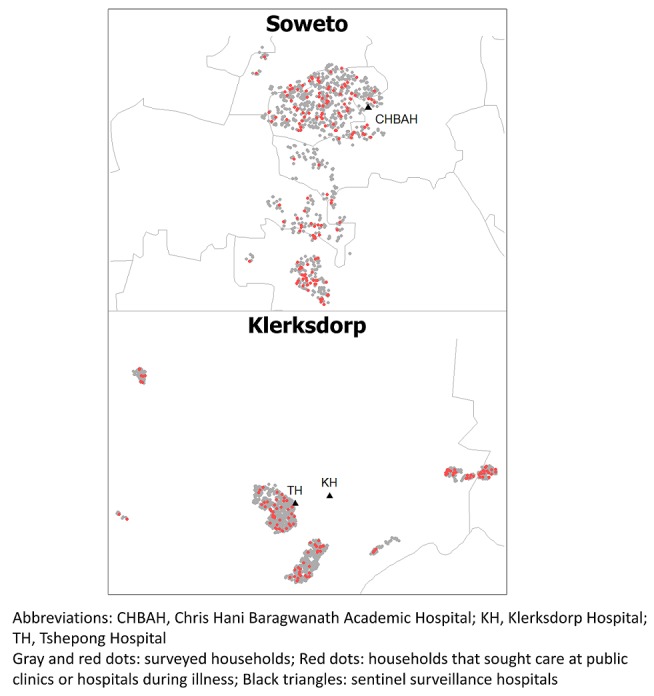
Household use of public clinics and hospitals for infectious syndromes-in Soweto and Klerksdorp, South Africa, 2011–2012

We compared patients who sought care for pneumonia at public versus private facilities and examined associations with the following characteristics: household monthly income, household size ≥ 4, sex, age < 5 years, and age < 18 years. Among pneumonia patients in Soweto who sought care with licensed medical providers, those who used public healthcare facilities (86/168, 51%) were more likely to have a household monthly income < ZAR 2000 (<237 USD [18]; OR: 2.9; 95% CI: 1.3-6.4) and to be HIV-infected (OR: 5.1; 95% CI 1.1-24.2) than those who used only private facilities. Among pneumonia patients in Klerksdorp who sought care with licensed medical providers, there were 50/94 (52%) who used public healthcare facilities; none of the characteristics examined were significantly associated with use of public versus only private facilities. In Klerksdorp, there were 57 individuals who met criteria for severe pneumonia, including 35 (61%) who survived. This includes 10 children < 5 years old, of whom 3 died. There were 23 (40%; 95% CI 29-53%) admitted to a hospital, and 17/20 (8%, 95% CI 64-95%) were admitted to the SARI sentinel hospital for Klerksdorp (Supplementary Table 5). The proportion of severe pneumonia patients in Soweto admitted to a hospital was similar (22/69, 32%, 95% CI 22-44%). There were 12 children < 5 years old with severe pneumonia in Soweto; none died. In Soweto, 9 people (50%, 95% CI 29-71%) were admitted to the Soweto sentinel hospital or one of its referral hospitals among the 18 individuals who specified the admitting hospital. Among 41 individuals in Klerksdorp who sought care at multiple facilities, the most common transition reported was from a public clinic to a public hospital (16/47, 34%; Figure 2) and people often consulted private doctors or general practitioners before or after consulting other facilities, including public healthcare facilities and pharmacies. In Soweto, the most common transition was also from the public clinic to the public hospital (18/71, 25%). People sought care at the public clinic before or after seeking care with several other types of providers, including hospitals and pharmacies. Consultation with public healthcare facilities was geographically widespread in both Soweto and Klerksdorp and did not appear to cluster around the sentinel hospitals (Figure 3). Among individuals who died, 25/56 (45%) in Klerksdorp and 7/29 (24%) in Soweto did not die in a hospital. The most frequent explanations for why an individual did not die in a hospital were that the person died suddenly or unexpectedly (Klerksdorp: 14/25, 56%; Soweto: 2/7, 29%) or that they died while trying to go to the hospital (Klerksdorp: 5/25, 20%; Soweto: 2/7, 29%). Other explanations for not dying in a hospital included not wanting to go (Soweto: 1), the hospital was too far away (Klerksdorp: 1), the person did not have any means of transportation to the hospital (Klerksdorp: 1), age (Klerksdorp: 1) and being recently discharged from the hospital (Klerksdorp: 1; Soweto: 1).

**Table 5:** Characteristics and healthcare seeking behavior of patients with severe pneumonia in last one year by site, in Soweto and Klerksdorp, South Africa, 2011–2012

	Klerksdorp n=57	Soweto n=69
Survived, n (%)	35 (61)	61 (88)
Admitted to hospital, n (%)	23 (40)	22 (32)
Hospitalized patients admitted to sentinel hospital, n/N (%)^a^	17/20 (85)	9/18 (50)
Age <5y, n (%)	10 (18)	12 (17)

a “Sentinel hospital” is KTHC for Klerksdorp and CHBAH for Soweto, and includes any referral hospitals. Percent reported out of those who specified the admitting hospital

## Discussion

We characterized healthcare utilization in two communities related to pneumonia, ILI, chronic respiratory illness, diarrhea in children < 5 years of age and meningitis. While public healthcare facilities were most commonly consulted for these syndromes, many residents in both communities consulted private healthcare providers and populations that use public versus private facilities differ. We characterized barriers to accessing healthcare with licensed medical providers and found that beliefs that their illness was not serious enough, perceived deficiencies in healthcare delivery and limited personal resources were reasons that people did not seek care. Public healthcare facilities were most commonly consulted for all infectious syndromes studied, but consultation with providers in the private sector, including private hospitals, clinics, and general practitioners, was also widespread. Our results complement prior studies among individuals with HIV, chronic non-infectious conditions and stroke, showing that private sector providers in South Africa are often consulted for care [11, 13, 33,34]. We found that in Soweto, pneumonia patients who consulted with public healthcare facilities were more likely to be poor and to self-identify as HIV-infected than pneumonia patients accessing the private healthcare sector. Pneumonia surveillance programs at Soweto public healthcare facilities may be less generalizable to higher-income residents and those who are not HIV-infected. These population characteristics must be considered when interpreting surveillance data, as the leading causes of respiratory infections, diarrhea and meningitis, differ by HIV status [35-38]. Consultations with traditional healers and religious leaders for infectious syndromes were relatively rare in our study compared to some prior surveys [13, 27, 34]. Pharmacies, which may advise customers according to both allopathic and complementary medicine traditions [39], were frequently used. Over a third of patients in this study who did not seek care with licensed medical providers did not believe their illness was serious enough, but several patients who did not seek care cited barriers to accessing healthcare. Many cited deficiencies in healthcare delivery or dissatisfaction with clinical services as reasons for not seeking care. Some patients mentioned medications being frequently out-of-stock; this has also been described in other areas of South Africa [9, 40]. Study participants also cited limitations in time, finances, or transportation options as reasons for not seeking care; these results are consistent with those from other provinces [40]. The barriers reported in this study indicate a healthcare system that still faces broad structural challenges, including social determinants of healthcare inequity, despite recent progress [41, 42]. Specific barriers to accessing care reported at each site can guide improvement of healthcare delivery to these communities. While this study focused on reasons that people did not seek care, it would also be important to know reasons for seeking care at different facilities, such as whether certain illness characteristics make people more likely to seek care.

The results of this study are subject to certain limitations. Some households did not participate because an adult caregiver could not be interviewed by the team or because the household declined participation; therefore, certain populations may not be represented by the study sample. The non-response rate also differed between study sites; we anticipated an overall non-response rate of approximately 15% in the study design and the actual non-response rate was 36% in Soweto and 11% in Klerksdorp. While efforts were made to represent all residential areas of the townships, including informal settlements, it is possible that transient populations or those living in non-residential areas, such as commercial districts, were systematically excluded from the sample. Because the case definitions were syndromic rather than laboratory-confirmed, they may have detected other etiologies, including other infectious or non-infectious causes of diseases. Because the case definition for meningitis included symptoms of chronic meningitis, which can be non-specific, it is likely that some illnesses were misclassified as meningitis. There was likely misclassification between acute and chronic, as well as mild and severe, respiratory illnesses by the case definitions, although the direction of potential bias from this is unclear. Certain case definitions required a recall period of up to one year by the patient or the patient's surrogate. Symptom reports decline with longer recall periods [43] and it is likely that more recent episodes and very severe episodes were more likely to be captured. Underlying conditions, including HIV, were likely underreported by individuals in this survey; the prevalence of self-reported HIV obtained in this study is much lower than estimates obtained among the general population or among pregnant women for both sites [21,22]. Data collection teams identified themselves as affiliated with the sentinel hospital at each site; this may have influenced respondents to report use of the sentinel hospital and they may have been less willing to report seeking care with non-licensed providers. Finally, the study sample size limited our ability to conduct analyses for healthcare-seeking behaviors by type of illness or certain age groups, because many of these syndromes are rare.

## Conclusion

Despite these limitations, this study describes healthcare-seeking patterns in two communities, providing context for surveillance data from public healthcare facilities [1, 20]; additionally, it identifies barriers to healthcare access among residents of public hospital catchment areas. Conducting interviews directly with community members in their homes through random geographic sampling can avoid some biases that affect health facility-based studies. When linked to surveillance data, these results will allow more accurate estimation of the burden of infectious respiratory illnesses, diarrhea and meningitis to improve targeting of prevention and treatment strategies in these communities.

### What is known about this topic

- Healthcare utilization varies regionally and is key to interpreting sentinel surveillance data;
- Understanding regional healthcare utilization, including barriers to accessing healthcare, informs efforts to improve access to and utilization of care.

### What this study adds

- In Soweto and Klerksdorp, private healthcare facilities, which may be missed by surveillance systems or government health interventions, were commonly used in addition to public healthcare facilities;
- Reasons that members of these communities did not seek care with licensed medical providers included medication shortages and limitations in time, finances and transportation.
